# Dishevelled Proteins Are Associated with Olfactory Sensory Neuron Presynaptic Terminals

**DOI:** 10.1371/journal.pone.0056561

**Published:** 2013-02-20

**Authors:** Diego J. Rodriguez-Gil, Wilbur Hu, Charles A. Greer

**Affiliations:** 1 Department of Neurosurgery, Yale University School of Medicine, New Haven, Connecticut, United States of America; 2 Department of Neurobiology, Yale University School of Medicine, New Haven, Connecticut, United States of America; Barnard College, Columbia University, United States of America

## Abstract

Olfactory sensory neurons (OSNs) project their axons from the olfactory epithelium toward the olfactory bulb (OB) in a heterogeneous and unsorted arrangement. However, as the axons approach the glomerular layer of the OB, axons from OSNs expressing the same odorant receptor (OR) sort and converge to form molecularly homogeneous glomeruli. Axon guidance cues, cell adhesion molecules, and OR induced activity have been implicated in the final targeting of OSN axons to specific glomeruli. Less understood, and often controversial, are the mechanisms used by OSN axons to initially navigate from the OE toward the OB. We previously demonstrated a role for Wnt and Frizzled (Fz) molecules in OSN axon extension and organization within the olfactory nerve. Building on that we now turned our attention to the downstream signaling cascades from Wnt-Fz interactions. Dishevelled (Dvl) is a key molecule downstream of Fz receptors. Three isoforms of Dvl with specific as well as overlapping functions are found in mammals. Here, we show that Dvl-1 expression is restricted to OSNs in the dorsal recess of the nasal cavity, and labels a unique subpopulation of glomeruli. Dvl-2 and Dvl-3 have a widespread distribution in both the OE and OB. Both Dvl-1 and Dvl-2 are associated with intra-glomerular pre-synaptic OSN terminals, suggesting a role in synapse formation/stabilization. Moreover, because Dvl proteins were observed in all OSN axons, we hypothesize that they are important determinants of OSN cell differentiation and axon extension.

## Introduction

Olfactory sensory neuron (OSN) axons face a challenging undertaking in establishing their final synapse location in the olfactory bulb (OB). Each OSN expresses 1 odorant receptor out of a repertoire of ∼1200 [1]. OSNs expressing the same odorant receptor are distributed through the olfactory epithelium (OE), intermingled with OSNs expressing different odorant receptors, but the axons from OSNs expressing the same odorant receptor converge in a reduced number of glomeruli (∼1–3 per OB). How this remarkable specificity is achieved remains controversial and accordingly, myriad molecules have been implicated in the process. Axon guidance molecules [2]–[6], cell adhesion molecules [7]–[9], extracellular molecules [10], [11], odorant receptors [12], [13], and odorant receptor induced electrical activity [14]–[16] have been shown to influence OSN axons and their specific glomerular location. A comprehensive model of how the OSN axons navigate and converge in a specific glomerulus has not yet emerged.

Wnts are secreted glycoproteins and Frizzleds (Fz) are 7 transmembrane receptors, together encoding for 29 proteins in the mammalian genome. First described as morphogens, members of the Wnt/Fz family are also implicated in axon guidance and synapse formation, among other processes. We and others previously described the expression pattern of several members of these families in the developing olfactory system [6], [17], and the existence of a subpopulation of olfactory ensheathing cells that activate the Wnt canonical pathway during development, and injury-induced regeneration [6], [17]–[19]. Moreover, we showed that addition of Wnt-5a to dissociated cultured OSNs induces significant changes in growth cone morphology and concomitant increases in neurite length leading us to suggest that Wnt-Fz interactions may be an important determinant of OSN axon extension [6].

Considering the variety of processes in which Wnt/Fz molecules are involved, and the evidence of promiscuous Wnt-Fz interactions (multiple Wnts can bind one Fz receptor), it is not surprising that the downstream signaling cascades following Wnt/Fz interactions are complex. They are divided into canonical (β-catenin dependent) and non-canonical pathways, although it is becoming increasingly clear that these pathways are not completely independent [20]. One key protein transducing the signal from the Fz receptor to downstream molecules is Dishevelled (Dvl) [21]. Three homologues of Dvl, with some degree of redundancy, have been described (Dvl-1, -2 and -3) [22]. To better understand Wnt-Fz signaling in OSNs we analyzed here the expression patterns and the subcellular distribution of all three Dvls in the developing mouse olfactory system. Dvl-1 expression is restricted to OSNs in the dorsal recess of the nasal cavity, and labels a unique subpopulation of glomeruli. Dvl-2 and -3 showed a widespread distribution both in the OE and the OB. Both Dvl-1 and Dvl-2 are associated with intra-glomerular pre-synaptic OSN terminals, suggesting a role in synapse formation/stabilization. Moreover, because Dvl proteins were observed in OSN dendritic knobs (Dvl-3) and in OSN axons (all of them) we hypothesize that they are important determinants of OSN cell differentiation and axon extension.

## Materials and Methods

The procedures and reagents used in this study were initially established and verified in our earlier analysis of the expression patterns of Wnts/Fzs in the olfactory system [6].

### Animals

Pregnant, time-mated CD-1 mice (Charles River, Wilmington, MA) were anesthetized with sodium pentobarbital (80 mg/kg, i.p.; Nembutal; Abbott Laboratories, North Chicago, IL) prior to cesarean section. The embryos collected at embryonic days (E) 13 and 17 (the day of conception is designated as E0) were fixed by immersion in 4% paraformaldehyde (PFA) in phosphate-buffered saline [PBS: 0.1 M phosphate buffer (PB) and 0.9% NaCl, pH 7.4] overnight at 4°C. Postnatal (P) mice at P4 were rapidly decapitated and immersion fixed in 4% PFA in PBS at 4°C overnight. For P7, P21 and adult tissue, mice were anesthetized with 80 mg/kg Nembutal and intracardiacally perfused with cold 4% PFA in PBS, pH 7.4. The heads were dissected, immersed in fixative for 2 h at 4°C, rinsed in PBS. Thy-1 YFP, GAD67-GFP and OMP-GFP mice were all previously described and bred in our colony [23], [24]. All procedures undertaken in this study were approved by Yale University’s Animal Care and Use Committee and conform to NIH guidelines.

### Sectioning

For immunohistochemistry, tissue was cryoprotected in 30% sucrose in PBS at 4°C until it sank and then was frozen in O.C.T. compound (Sakura Finetek, Torrance, CA). Coronal sections, 20 µm thick, were obtained using a Reichert-Jung 2800 Frigocut E cryostat. Sections were thaw mounted onto SuperFrost Plus microscope slides (Fisher Scientific, Pittsburgh, PA), air-dried, and stored at −20°C until use.

### Immunohistochemistry

Tissue was thawed, air-dried and processed for an antigen retrieval protocol when needed. Antigen retrieval was similar to a previously published protocol [25]. Briefly 0.01 M sodium citrate was heated to 70°C followed by immersion of the slides in the solution for 30 min. Tissue integrity was better preserved using this protocol and results were the same as the previously described steaming protocol [6]. Tissue was then incubated with 2% bovine serum albumin (BSA) (Sigma, St. Louis, MO) in PBS-T (PBS with 0.3% Triton X-100, Sigma) for 30 min to block nonspecific binding sites. Incubation with primary antibodies diluted in blocking buffer was performed overnight at room temperature (RT). Sections were then washed 3 times in PBS-T for 5 min and incubated in secondary antibodies conjugated to Alexa Fluor (Molecular Probes, Eugene, OR) diluted 1∶1,000 in PBS-T for 1 h at RT. Sections were washed in PBS-T, rinsed in PBS, mounted in Fluoro-Gel mounting medium (Electron Microscopy Sciences, PA), and coverslipped.

Images were acquired with a Leica confocal microscope. Each color channel was obtained sequentially to prevent side-band excitation of the different fluorophores. Where indicated, Z-stack images were acquired at 0.5 µm steps and maximum projections, generated with Leica confocal software are shown. Digital images were color balanced using Adobe Photoshop 6.0 (Adobe Systems, San Jose, CA). The composition of the images was not altered in any way. Plates were constructed using Corel Draw 12.0 (Corel, Ottawa, Ontario, Canada).

To analyze the expression levels of Dvl-1, OCAM and NQO1, we traced a line connecting all the glomeruli using Image J. On each OB section the line started in the most ventral part and continued connecting the glomeruli in the lateral, dorsal and medial wall of the OB ending in the same place it started. The relative optical density (in arbitrary units) of each channel was determined and plotted. Background staining was subtracted, using the inter-glomerular signal as background.

### Immunoelectron Microscopy

For electron microscopy, the mice were perfused with 4% PFA and 0.5% glutaraldehyde, followed by postfixation in the same fixative for 2 h. Brains were rinsed in PBS overnight and cut on a vibratome (50 µm). Endogenous peroxidases were blocked by incubation in 0.3% H_2_O_2_ in PBS. Sections were blocked in 2% BSA at RT for 30 min and incubated with primary antibody overnight at 4°C. The biotinylated secondary antibody was followed by the ABC reagent (Vector) and a DAB peroxidase reaction. Tissue was then counterstained with osmium tetroxide and embedded in EPON for thin sectioning. Sections of 70–100 nm were examined on a Phillips transmission electron microscope and photographed at primary magnifications of 5,000–15,000X.

### In situ Hybridization

*In situ* hybridization was done following an established protocol [26]. The probe for Dvl-1 was generated by PCR from total olfactory epithelium RNA from postnatal day 4 mice. Primers were as follows: 5′-GACCGCATGTGGCTTAAGAT-3′ and 5′-TGGCGGCTACCTGTAAGTTC-3′, with the addition of T7 or T3 polymerase sequences for RNA synthesis of sense and antisense probes, respectively. This Dvl-1 sequence was chosen because its identity with the other two Dvl molecules less than 80%, allowing us to differentiate between Dvl-1 and Dvl-2, -3 [27]. The amplified product was sequenced and used to generate a DIG-labeled RNA probe. *In situ* hybridization was developed with HNPP/Fast Red (Roche).

### Western Blot

Western blot experiments were performed following an established protocol from our lab [16].

### Antibodies

The following antibodies were used: mouse anti Dvl-1 (Santa Cruz, CA, sc-8025) at 1/200 with antigen retrieval; rabbit anti Dvl-2 (GeneTex, TX, GTX103878) at 1/250; rabbit anti Dvl-3 (GeneTex, TX, GTX102509) at 1/250; goat anti NQO1 (Abcam, MA, ab2346) at 1/3000; rabbit anti OCAM (gift from Dr. K. Mori) at 1/1000; rabbit anti VGlut2 (Synaptic Systems, Germany, 135403) at 1/2000; rabbit anti Synaptotagmin 1 (Synaptic Systems, Germany, 105002); rabbit anti Synaptophysin (Dako, CA, A0010). Secondary antibodies were Alexa-Fluor conjugated (Invitrogen) at 1/1000 and Cyanine Dye conjugated (Jackson ImmunoResearch) at 1/300. Nuclei were counterstained with DRAQ5 (Biostatus, Leicestershire, UK).

Dvl-1 antibody has been extensively used in Western blots (e.g. [28]–[34])) as well as immunohistochemistry ([35]) experiments. Nonetheless, due to the intriguing pattern observed in the olfactory system, we performed the following specificity controls. COS7 cells were transfected with a Dvl-1-Flag tag plasmid (generous gift from Dr. Dianqing Wu, Yale University), and subjected to immunocytochemistry or Western blot analysis. Immunocytochemistry showed a complete colocalization of the staining (Figure S1A) for Dvl-1 and Flag tag (F1804, Sigma) while Western blot analysis showed a single band of the expected size (Figure S1B, lane 2). After transfecting these cells with Dvl-1 and a previously published Dvl-shRNA sequence [36], we detected a decrease in Dvl-1 expression (Figure S1B, lane 4 and Table S1). To further test the specificity in mouse brain sections, we stained sections from the cortex and cerebellum and obtained identical staining patterns (data not shown) to those previously reported [37]. The expression pattern of Dvl-2 and Dvl-3 antibodies matched published *in situ* hybridization data in the olfactory bulb ([38]), and our own *in situ* hybridization data for Dvl-2 (data not shown). The immunogens used to generate Dvl-2 and Dvl-3 antibodies show a 99% and 97% of identity with their mouse homologue respectively.

## Results

We previously identified two Fz receptors (Fz-1 and Fz-3) expressed in OSNs during embryonic development and in the adult mouse. Both Fz-1 and Fz-3 were localized in OSN cell bodies, dendrites, knobs, cilia and axons [6]. It seems plausible that the signaling cascade initiated after Fz receptor activation is dependent on the subcellular distribution of the downstream pathway molecules. To identify candidate Fz signaling sites in OSNs, we analyzed the subcellular distribution of the 3 Dvl homologues, a key molecule transducing the signal from Fz receptors to downstream proteins. All three Dvl molecules were expressed in OSNs. Nonetheless, we focused largely on Dvl-1 expression because it showed the most intriguing distribution pattern.

### Dishevelled 1

**Figure 1:**
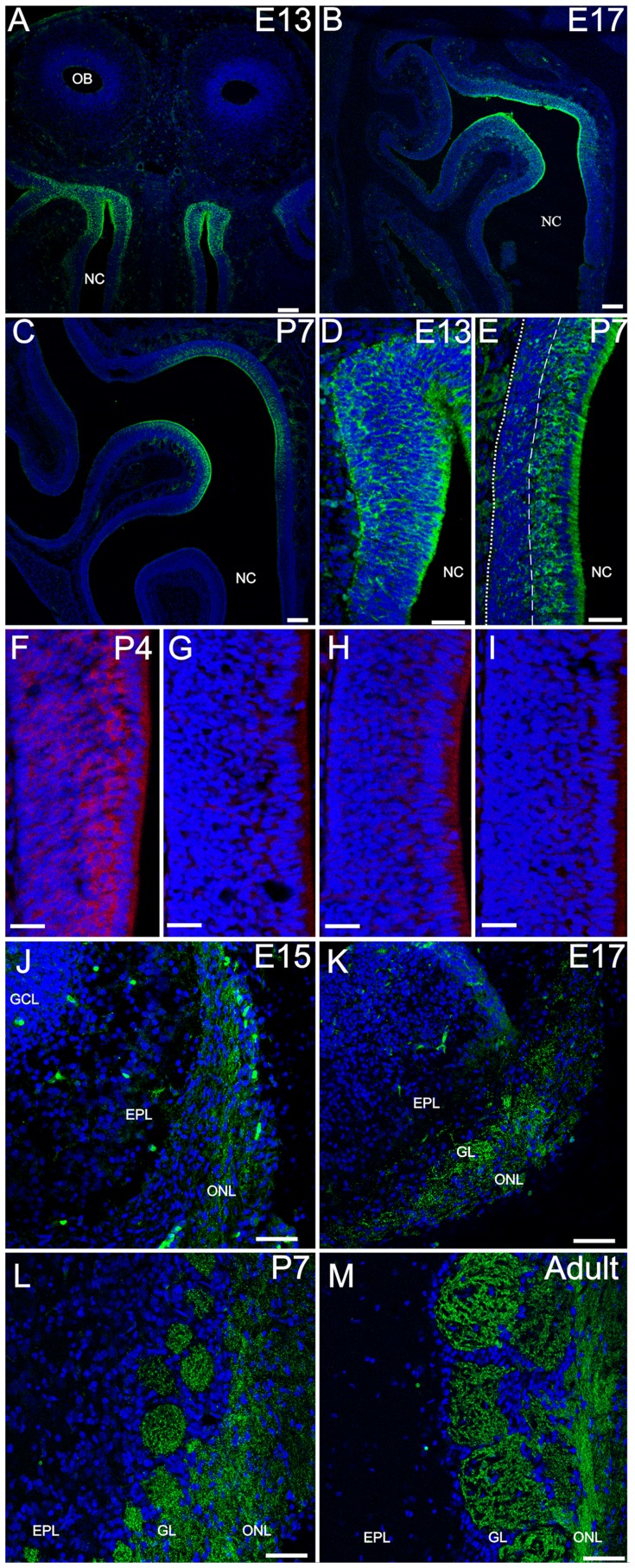
Dvl-1 is expressed in zone 1 in the olfactory epithelium and restricted to the ONL and GL in the olfactory bulb. Dvl-1 (green) is expressed as early as E13 (A) throughout the OE and it expression remains during embryonic and early postnatal development (B, C). By P7, mature olfactory sensory neurons and sustentacular cells show the higher levels of expression in the OE. D, E: Higher magnification images of E13 and P7 respectively. Dotted line marks the basal membrane of the OE; dashed line demarks the lower edge of Dvl-1 expressing OSNs. Dvl-1 *in situ* hybridization showed the same expression pattern at P4 in zone 1 (F) with no expression outside this zone (G). As expected, control sense probe did not show any staining in zone 1 (H) or outside (I). Dvl-1 was detected in the OB by E15 (J) in the ONL, and it extended to the developing GL by E17 (K). The expression remained in the ONL and GL after birth and was observed also in adult animals (L, M). Due to the lack of perfusion, at E15 and E17 an intense immunoflourescence could be detected in blood cells (J, K). Nuclei were counterstained with DRAQ5 (blue). EPL: external plexiform layer; GCL: granule cell layer; GL: glomerular layer; NC: Nasal cavity; NL: olfactory nerve layer; OB: Olfactory bulb. Scale bar = 100 µm (A–C), 50 µm (D–M).

Dvl-1 was expressed in the OE as early as embryonic day (E) 13 (Figure 1A). Expression was restricted to the dorsal recess of the OE and remained so during postnatal development (Figure 1A, B, C). During embryonic development all cells throughout the basal-apical extent of the OE showed Dvl-1 expression; after birth Dvl-1 showed higher levels of expression in the apical layer of the OE occupied by mature OSNs (compare Figure 1D and E). This specific expression pattern was confirmed with *in situ* hybridization (Figure 1F–I). The antisense probe showed expression in zone 1 (Figure 1F) and not in any other zones of the OE (Figure 1G), while the sense probe showed no staining in either zone 1 (Figure 1H) or outside (Figure 1I). In the OB, Dvl-1 was detected only after E15 in the developing olfactory nerve layer (ONL) (Figure 1J), consistent with the first axons penetrating the dendritic zone and the first formation of OSN synapses [39], [40]. By E17 Dvl-1 expression was evident in the ONL and glomeruli (Figure 1K), and continued so during the immediate postnatal period (Figure 1L) and into the adult (Figure 1M). Because of the restricted expression pattern observed in the OE, we analyzed the distribution of Dvl-1 expressing glomeruli in the OB. In the most anterior part of the OB, dorso-lateral glomeruli showed Dvl-1 expression while posterior sections showed dorso-medial Dvl-1 positive glomeruli (Figure S2).

**Figure 2:**
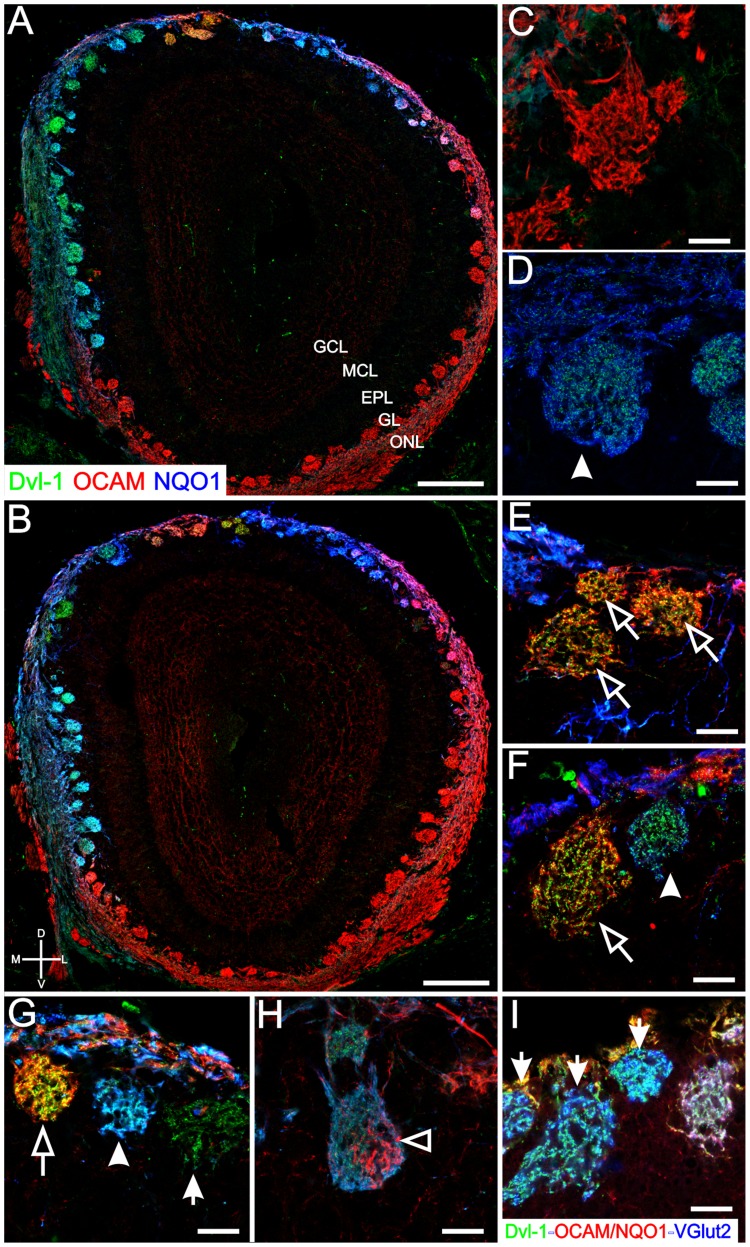
Dvl-1 is expressed in a unique subpopulation of glomeruli. A, B: Dvl-1 expression in the OB was analyzed in the context of two classical markers: NQO1 (zone I/dorsal OE) and OCAM (zones I-IV/ventral OE). All NQO1-expressing glomeruli showed Dvl-1 expression (A, B, D, F, G, H). A few OCAM-expressing glomeruli located in the dorsal part of the OB, also showed Dvl-1 expression (A, B, and open arrows in E, F, G). In the ventral part of the OB, only OCAM-expressing glomeruli were observed (A, B, C). In the transition NQO1/OCAM zone, those glomeruli expressing both markers, showed expression of Dvl-1 only in the NQO1 positive axons (A, B, and open arrowhead in H). Dvl-1 also revealed a subset of glomeruli negative for both classical markers (A, B, and arrows in G, I). In order to test whether these only Dvl-1 expressing glomeruli are functional, we stained for VGlut-2 (blue), Dvl-1 (green) and OCAM+NQO1 (both in red) (I). These glomeruli expressed VGlut-2 (arrows), consistent with functional activity. Scale bar  = 200 µm in A, B; 20 µm C–I. ONL: olfactory nerve layer; GL: glomerular layer; EPL: external plexiform layer; MCL: Mitral cell layer; GCL: granule cell layer.

The expression pattern of Dvl-1 is reminiscent of the previously described zone 1 in the OE [41]. Zone 1 is characterized by the expression of class I odorant receptors and is also distinguished by the expression of the markers NQO1 (NAD(P)H dehydrogenase, quinone 1) [42] and OMACS (olfactory-specific medium-chain acyl CoA synthetase) [43]. A few class II odorant receptors and some TAARs (trace amine-associated receptors) are also expressed in zone 1 [44], [45]. NQO1 labels glomeruli located in the dorso-medial OB. The ventral zones in the OE (zones 2–4), are characterized by the expression of class II odorant receptors and are recognized by the expression of OCAM (olfactory cell adhesion molecule) [46]. OCAM labels glomeruli located in the ventro-lateral OB. We therefore analyzed the distribution of Dvl-1 expressing glomeruli in relation to two of these classical markers, NQO1 and OCAM, to test the hypothesis that Dvl-1 expressing glomeruli will show the same pattern of expression as NQO1. Figure 2A and B show in sections from two different antero-posterior positions, that the expression patterns at postnatal day 4 of NQO1 (blue) and OCAM (red) are mutually exclusive, and that the Dvl-1 (green) expression colocalizes predominately with NQO1 and not with OCAM. It is also of interest that the expression levels of these three markers differ across glomeruli as evidenced by the diverse colors observed largely in the dorso-medial walls of the OB. Further analyses of individual glomeruli showed, as expected, that Dvl-1 is not expressed by any ventro-lateral OCAM+ glomeruli (Figure 2C), and Dv1-1 is expressed by all dorso-medial NQO1+ glomeruli (arrowhead in Figure 2D, F, G). In the OCAM/NQO1 transition area, those glomeruli co-expressing OCAM and NQO1, showed expression of Dvl-1 only in the NQO1 OSN axons (open arrowhead in Figure 2H). Analysis of individual glomeruli showed some surprising results. First, a subset of ectopically located OCAM+ glomeruli found in the most dorsal part of the OB (open arrows in Figure 2E, F, G) express Dvl-1 and second, we identified a few dorso-medial glomeruli that express only Dvl-1 and neither of the other two classical markers, OCAM or NQO1 (arrows in Figure 2G, I). These characteristic glomeruli were also observed in all of the OB sections from adult mice (data not shown), suggesting that these expression patterns are stable and not restricted to early postnatal development.

**Figure 3:**
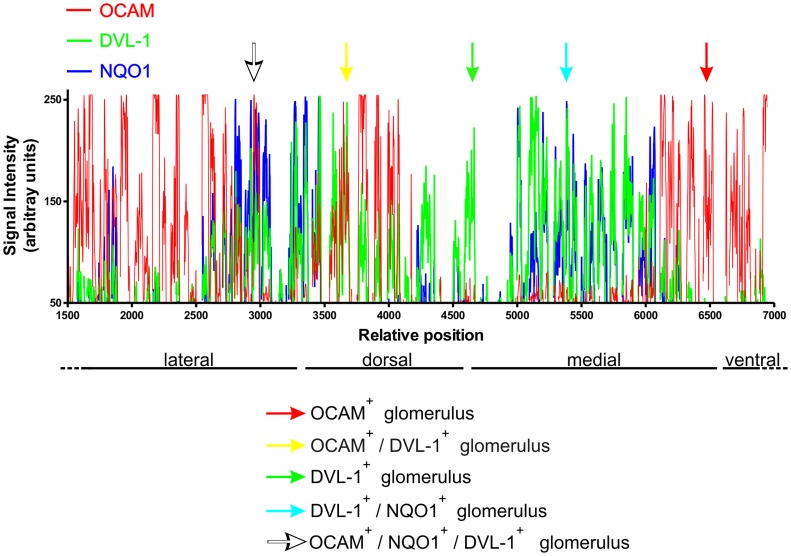
Tracing analysis also showed Dvl-1 only expressing glomeruli, predominately in the dorso-lateral and dorso-medial domains. The ventral domain was not included in order to increase clarity and emphasize the glomeruli expressing multiple markers. This analysis identified OCAM+ glomeruli (red arrow), OCAM+/Dvl-1+ glomeruli (yellow arrow), Dvl-1+ glomeruli (green arrow), NQO1+/Dvl-1+ glomeruli (turquoise arrow), OCAM+/NQO1+/Dvl-1+ glomeruli (white arrow). The X axis represents the line connecting all the glomeruli, stretched out linearly. We removed most of the ventral and lateral tracing (relative position 0–1500) because all these glomeruli were only OCAM positive.

We then analyzed signal intensity of all three markers around the circumference of the OB by tracing a line connecting all the glomeruli, and determined the relative optical density (fluorescence) for each of the antibodies. As shown in Figure 3, individual glomeruli had a characteristic appearance distinguished by the relative optical density for each of the three antibodies. Single labeled glomeruli, OCAM (red arrow), or Dvl-1 (green arrow); double labeled glomeruli (OCAM/Dvl-1, yellow arrow), or NQO1/Dvl-1 (cyan arrow); and triple labeled glomeruli (OCAM/NQO1/Dvl-1, white arrow) were evident. Finally, we asked if the Dvl-1 only expressing glomeruli were functional. To answer this we used VGlut-2 (vesicular glutamate transporter 2), which is expressed in OSN neurons and is associated with active glomerular synapses. To recognize the Dvl-1 only expressing glomeruli, we stained the OB with NQO1 and OCAM and developed both in the same color (red) leaving the other two channels for Dvl-1 (green) and VGlut2 (blue) (Figure 2I). As seen in Figure 2I, glomeruli that expressed only Dvl-1, and did not express either OCAM or NQO1, still expressed VGlut2 (arrows in 2I) suggesting that Dvl-1 only glomeruli are functionally active.

**Figure 4:**
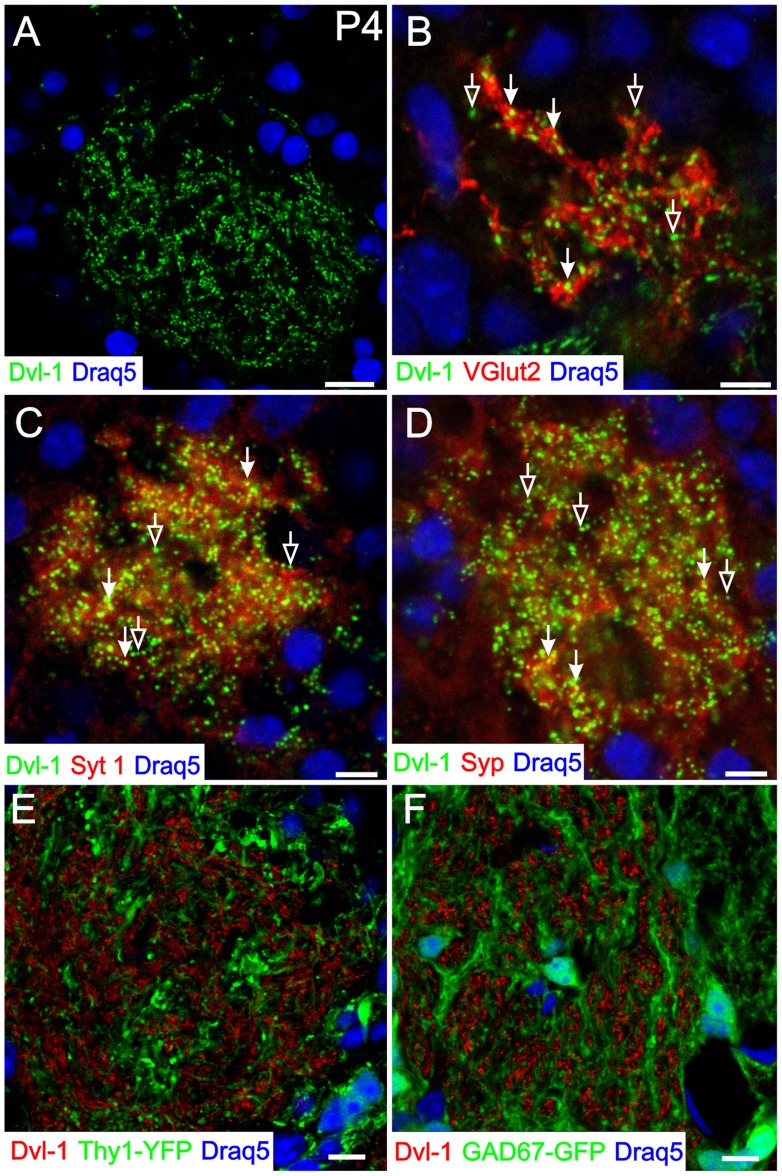
Dvl-1 exhibits a punctate distribution in OSN axons, and is associated with synapses. In order to test if the punctate distribution of Dvl-1 (A) is associated with synapses, we double stained for VGlut2 (B), synaptotagmin 1 (Syt 1, C), and synaptophysin (Syp, D) three classical presynaptic terminal markers. All three synaptic markers co-localized with Dvl-1 puncta (filled arrows in B–D). However, there were instances in which a Dvl-1 puncta did not colocalize suggesting that the expression of synaptic markers may vary among OSN terminals (open arrows in B–D). To rule out the expression of Dvl-1 in other populations of cells with glomerular localization we used GAD-67 GFP mice to assess periglomerular cells and Thy-1-YFP mice to assess mitral cells (E, F, respectively). Dvl-1 puncta were not present in any GAD-67 GFP or Thy-1 YFP processes, consistent with the notion that Dvl-1 is only in OSN axons in the glomeruli. Nuclei were counterstained with DRAQ5 (blue). Scale bar  = 10 µm in A, E, F; 5 µm in B–D.

Dvl-1 has a punctate expression (Figure 4A) and as noted above, colocalizes with VGlut2. Because Dvl proteins have been implicated in synapse formation elsewhere in brain [47] we extended our analysis of Dvl-1 and pre-synaptic markers. When we analyzed in detail the expression of Dvl-1 and VGlut2, we found that not all the Dvl-1 puncta colocalized with VGlut2 (Figure 4B). Similarly, neither synaptotagmin 1 (Syt 1) nor synaptophysin (Syp), both of which are presynaptic proteins found in both OSN axons and glomerular dendritic presynaptic specializations [48], colocalized with all Dvl-1 puncta (Figure 4C, D). This lack of complete colocalization could be due to an extrasynaptic expression of Dvl-1 in OSN terminals, or to the expression of Dvl-1 in other cell types in the OB that do not express any of the three presynaptic markers we tested. To determine if other cell types in the OB express Dvl-1 we used two mouse lines. One transgenic line expresses YFP under the control of the Thy-1 promoter and selectively labels mitral and tufted cells in the OB [49]. The second line expresses GFP under the control of GAD-67 (Glutamic acid decarboxylase 67) and labels GABAergic interneurons in the OB [50]. When we stained sections from these two mouse lines for Dvl-1, we never observed Dvl-1 puncta in projection neurons or interneurons (Figure 4E, F), suggesting that only intraglomerular OSN axon terminals express Dvl-1. In those instances in which we do not find colocalization with presynaptic markers it seems reasonable to suggest that there is an extra-synaptic expression of Dvl-1 associated with OSN terminals. Alternatively, it may reflect axons that are still in an immature, unstable, phase of development or perhaps moving toward degeneration and turnover.

### Dishevelled 2

**Figure 5:**
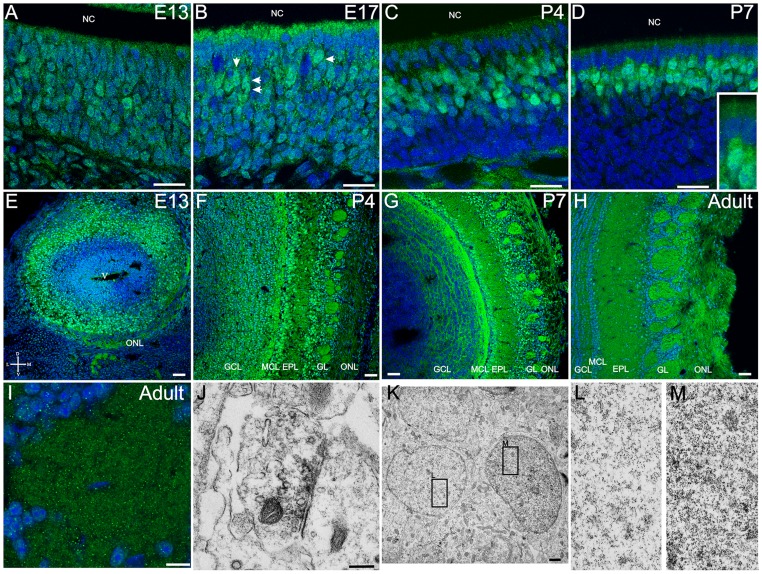
Dvl-2 is expressed in OSN, in all layers through the olfactory bulb and is associated with synapses inside the glomeruli. Dvl-2 is expressed as early as E13 (A) throughout the OE. During embryonic development some cells showed higher levels of expression than others (B, arrows) and after birth, Dvl-2 expression was restricted to the mature OSN layer of the OE (C, D). Dvl-2 expression was not restricted to the cytoplasm, but was also detected in the nucleus (D, inset). In the developing OB, Dvl-2 was observed as early as E13 (E), and it was detected up to adult (F–H). Even though Dvl-2 was detected in all layers, levels of expression differed between layers and in between animals of the same age (especially after birth). Glomeruli showed a punctate distribution of Dvl-2 (I) similar to that observed for Dvl-1. Dvl-2 expression was not restricted to the cytoplasm and processes, but it also showed nuclear expression, although this expression was not observed in all cells of any given cell type. Electron microscopy images of Dvl-2 stained sections showed that inside the glomerulus, Dvl-2 is in the presynaptic terminal (J). K: In agreement with the immunofluorescence, electron micrographs of periglomerular cells showed some nuclei with Dvl-2 expression (M), while others showed no evidence of Dvl-2 expression (L). Nuclei were counterstained with DRAQ5 (blue). Scale bar  = 20 µm in A–D; 50 µm in E–H; 10 µm in I; 200 nm in K; 1 µm in K. EPL: external plexiform layer; GCL: granule cell layer; GL: glomerular layer; MCL: Mitral cell layer; NC: Nasal cavity; OE, olfactory epithelium; OB: Olfactory bulb; ONL: olfactory nerve layer; V: ventricle.

Dvl-2 was observed throughout the basal-apical extent of the OE at E13 (Figure 5A) with some variability in the level of expression across cells. This differential expression in OSNs became more marked towards the end of the embryonic development at E17 (Figure 5B). By P4, Dvl-2 expression was largely limited to the more superficial aspects of the OE, the sublaminae generally associated with mature OSNs (Figure 5C, D). Of interest, Dvl-2 expression was not restricted to the cytoplasm, it was also observed inside the nucleus suggesting the possibility of a role in regulating transcription (Figure 5D inset). Dvl-2 did not show the regional or zonally restricted pattern of expression in the OE that was found for Dvl-1. Rather, it appeared uniformly throughout the OE (i.e. Dvl-2 was observed in all four zones in the OE).

In the OB, Dvl-2 showed a widespread expression pattern, with all layers showing Dvl-2 expression (Figure 5E–H). From E13 to P7 most cell bodies showed heavy staining throughout the OB (Figure 5E–G). However, in the adult the number of Dvl-2 positive cell bodies appears decreased, with staining most prominent in the neuropil of the glomeruli and EPL (Figure 5H). The expression of Dvl-2 within the glomerular neuropil was similar to the punctate pattern seen with Dvl-1 (Figure 5I). Immunoelectron-microscopy demonstrated that Dvl-2 is present in the presynaptic OSN terminals inside the glomerulus (Figure 5J), once again suggesting that Dvl proteins are involved in synaptic connectivity in the OB glomeruli. In the OB, like the OSNs, Dvl-2 was observed inside the nucleus although not all the cells showed this expression pattern (Figure S3). Immunoelectron-microscopy clearly shows that adjacent cells can differ in their nuclear expression of Dvl-2. In Figure 5K, M nuclear expression of Dvl-2 is evident while it is not present in the neighboring cell shown in Figure 5K, L.

### Dishevelled 3

**Figure 6:**
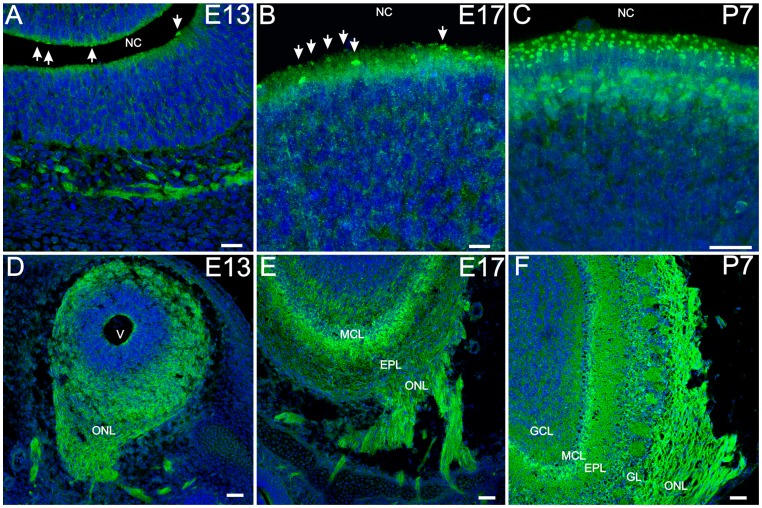
Dvl-3 is expressed in OSN knobs and in all layers of the olfactory bulb. Dvl-3 expression levels in OSN cell bodies remain low during embryonic development (A, B) and increase after birth (C). Dvl-3 was detected in OSN knobs (arrows in A – B), but only those knobs expressing levels higher that those of sustentacular cells were readily detectable. Z-stack projections (B, C) showed higher numbers of Dvl-3 expressing knobs. In the olfactory bulb Dvl-3 was observed through all the layers during embryonic development and postnatally (D – F). Nuclei were counterstained with DRAQ5 (blue). Scale bar = 20 µm in A, C; 10 µm in B; 50 µm in D–F. AOB: accessory olfactory bulb.

Finally, we analyzed the expression of Dvl-3. In the OE, Dvl-3 was observed in OSN dendritic knobs (Figure 6A–C), and at very low levels in OSN cell bodies. Dvl-3 was uniformly expressed without evidence of zonal or regional expression in the OE, sharing this aspect with Dvl-2 expression and in opposition to Dvl-1. By P7 it is evident that Dvl-3 is largely in the more superficially located mature OSNs cell bodies, but even at E13 and E17 it is apparent that immature OSNs show little evidence of being Dvl-3 positive. Sustentacular cells showed high levels of expression, mostly during embryonic development, and because of this identification of individual OSN dendritic knobs was difficult in single confocal images. Dvl-3 expression was higher in OSN dendritic knobs and the number of Dvl-3 expressing knobs increases during development (Figure 6A, B, C). In the OB, the expression pattern of Dvl-3 was very similar to that of Dvl-2, except for the lack of expression of Dvl-3 inside cell nuclei. All cell layers showed Dvl-3 expression, with higher levels in the ONL and the mitral cell layer (Figure 6D–F).

## Discussion

Secreted Wnts play fundamental roles in diverse processes including cell polarization, migration, differentiation, and axon extension. Several members of the Wnt family and their seven transmembrane receptors, Fz, are expressed in the olfactory system suggesting a role during development [6], [17], [18], [51]. In one example, we have shown that Wnt-5a induces OSN axon extension in a dose-dependent manner [6]. However, Wnt/Fz signaling can occur via several alternative cascades and which of those may be active in OSNs is not known. An exception is found among a subset of olfactory ensheathing glia that in transgenic mice provide evidence of using the canonical Wnt signaling pathway during early development and following lesion-induced regeneration [6], [17], [18].

To begin dissecting Wnt/Frizzled signaling in the olfactory system, we studied the expression of Dvl molecules. Dvl is a key signaling molecule downstream of Frizzled receptors, and is a scaffold protein necessary to several of the proposed signaling cascades. Three Dvl homologues have been described both in humans and mice (Dvl-1, -2 and -3) and all of them present conserved domains: the DIX domain (Dishevelled, Axin), the PDZ domain (Postsynaptic density 95, Discs Large, Zonula occludens-1) and the DEP domain (Dvl, Egl-10, Pleckstrin) [21]. The DIX domain is responsible for the polymerization of Dvl proteins, which appear as cytoplasmic puncta [52]. In agreement with this, we found a punctate distribution of Dvl proteins in the OB glomeruli. Moreover, we were able to demonstrate that a significant proportion of these puncta colocalize with presynaptic markers in OSN terminals. Although we did not determine the subcellular compartment of Dvl puncta in OSN axons in the olfactory nerve layer, it has been shown that Dvl can bind to microtubules and, by inhibiting GSK3β can increase microtubule stability [53]. Therefore, we speculate that Dvl puncta in OSN axons is associated with stabilization of growing microtubules.

Target recognition and synapse formation have been attributed to several classes of cell adhesion molecules, including cadherins, neuroligin, neurexin and Syncam, all of which act when the axons and the dendrites are in close proximity. Prior to close apposition of processes secreted molecules such as semaphorins, netrins and Wnts act in axon guidance and as priming factors in the process of synapse formation [54]. In the cerebellum, Wnt-7a regulates the maturation of the synapses between the mossy fibers and the granule cells, a process mediated by signaling through Dvl-1 [47]. In the olfactory system, Wnt-7a is expressed by interneurons and mitral cells [51], while Dvl-1 is expressed in a subset of olfactory sensory neuron axons and is associated with presynaptic terminal markers. It is possible then, that a similar role is played by these molecules in the formation of the connections between OSN axons and mitral cell dendrites. Fz-7 has been shown to mediate Wnt-7a effects in muscle [55], and in the hippocampus Fz-5 mediates Wnt-7a synaptogenic effect [56]. Interestingly, none of these Fz receptors are observed in OSN axons. Nonetheless, given that Wnt-7a and Wnt-7b are 95% identical [17] and Wnt-7b interacts with Fz-1 [57] it is possible to speculate that in the OB Wnt-7a activates Dvl-1 through Fz-1 [6].

There are multiple lines of evidence linking the primary cilium and Wnt signaling. The majority of the data suggest that the cilium and the basal body exert an inhibitory effect on the canonical Wnt signaling pathway [58]–[60] possibly promoting the activation of non-canonical pathways. We have shown that Fz-1 and Fz-3 are expressed in OSNs including the cilia and the dendritic knob [6]. We now have evidence of Dvl-1 and Dvl-3 expression in OSN cell bodies and apical dendrites. In particular, Dvl-3 expression was very strong in the dendritic knob where the ciliary basal bodies are located. Because the canonical Wnt pathway is not active in OSNs [6], [18], it is tempting to speculate that Dvl-3 expression in OSN dendritic knobs activates a Wnt-mediated down-regulation of the canonical pathway via the cilia and basal bodies. Interestingly, suppression of some Bardet-Biedl syndrome (BBS) proteins *in vitro* results in increased activation of canonical Wnt signaling [61]. BBS proteins, as well as other cilia associated proteins, have been described in the olfactory system, and suppression of these proteins resulted in the absence or malformation of sensory cilia [62]–[64]. Down-regulation or loss of the olfactory cilia under these conditions would provide an interesting model to determine if there is a corresponding imbalance between the canonical and non-canonical Wnt signaling pathways in OSNs.

Our findings on Dvl-1 expression have particular significance to a possible role of Wnt signaling and topography in the olfactory pathway. First, we identified some glomeruli that were positive for Dvl-1 and negative for both NQO1 and OCAM. These glomeruli, which are functional based on the expression of VGlut2, may have been overlooked in the past using these two markers. This finding adds to the complexity observed in the olfactory bulb, where it seems that each glomerulus is unique considering not only the expression of only one odorant receptor, but also for the graded expression of specific sets of adhesion-related molecules [7], [8]. Second, Dvl-1 expression was restricted to the most dorsal recess of the nasal cavity. This area has been described as zone 1 [41], [65], [66] and it is characterized by the expression of largely Class I odorant receptors [67]. OSNs located in this area also express specific markers such as NQO1 and OMACS and do not generally express OCAM. However, we found several clustered OCAM positive glomeruli in the midst of the otherwise OCAM negative dorso-medial region of the OB. The glomeruli were consistent across subjects and ages, and also co-expressed Dvl-1, which is normally restricted to OCAM negative glomeruli. This suggests that there is a subgroup of olfactory sensory neurons sharing characteristics of different zones (i.e. Dvl-1 expression and dorsal projection in the olfactory bulb, characteristics of sensory neurons located in the dorsal recess of the nasal cavity, and expression of OCAM, characteristic of the ventral zones). It may be that the OSNs projecting to this small cluster of glomeruli are: 1) Class II odorant receptor expressing sensory neurons located in zone 1 [44]; 2) that these sensory neurons are located at the interface between zone 1 and 2 in agreement with the proposed overlapping continuum of zones [68]; 3) it has been recently shown that TAARs neurons [45] project their axons to dorsally located glomeruli [69] and that these are OCAM positive [70].

In summary, we showed that Dvl molecules are expressed in the olfactory system, and based on their distribution and temporal expression patterns we speculate that they are involved in multiple processes such as controlling signaling from olfactory sensory neuron cilia, axon guidance and synapse formation/stabilization. In the future it will be interesting to analyze the downstream effectors of Dvl in olfactory sensory neurons to elucidate which specific Wnt/Fz signaling pathways are used by olfactory sensory neurons.

## Supporting Information

Figure S1 **The antibody sc-8025 recognizes Dvl-1 protein.** A: COS7 cells were transfected with a Dvl-1 Flag-tagged construct (top) or a control plasmid (bottom) and stained for Dvl-1 (green) and Flag (red), and counterstained for nuclei with Dapi (blue). 100% colocalization was observed between both markers in transfected cells. B: COS7 cells were transfected with the same Dvl-1-Flag plasmid (lanes 2 and 4) in the presence of a Dvl-shRNA (lanes 3 and 4). Homogenized cells were subjected to Western blot analysis and developed with anti-Dvl-1 and anti-β-actin (Abcam) antibodies. Only one specific band of the expected size was observed after transfection with Dvl-1 (lanes 2 and 4) and a reduction of the expression after co-transfection with the Dvl-shRNA (lane 4). Expression levels were standarized dividing Dvl-1 signal by β-actin signal and are shown in Table S1.
(TIF)

Figure S2 **Dvl-1 expression shifts from anterior-lateral to posterior-medial.** In agreement with the expression observed in the OE, Dvl-1-expressing axons were observed in a restricted set of glomeruli. At P4 in the most anterior OB sections, dorso-lateral glomeruli showed Dvl-1 expression while posterior sections showed dorso-medial glomeruli Dvl-1 expression. White dashed lines demark where positive Dvl-1 glomeruli were observed.
(TIF)

Figure S3 **Dvl-2 is observed in OSN axons and dendritic processes in the glomeruli.** Most of Dvl-2 puncta was observed in OSN axon, as evidenced for the colocalization in OMP-GFP expressing mice (circle in A).Nonetheless, some of them did not colocalized (dashed circle in A). To analyze which cell type expressed these puncta, we stained for Dvl-2 in Thy1-YFP (to label projection neurons, B) and GAD-67-GFP (to label inhibitory interneurons, C), and in both cases we were able to detect some colocalization (arrows in B and C). Expression of Dvl-2 in the OB of GAD-67-GFP mice showed higher levels of colocalization in the EPL (D, E) than in the glomerular layer (D, F). D: green channel was removed in the left part of the image to show the difference in Dvl-2 expression. Nuclei were counterstained with DRAQ5 (blue). Scale bar  = 10 µm in A-C, E, F; 20 µm in D.
(TIF)

Table S1 (DOC)
