# Supplementary figures and images for: Dishevelled Proteins Are Associated with Olfactory Sensory Neuron Presynaptic Terminals
Source: PLoS One. 2013 Feb 20;8(2):e56561. doi: 10.1371/journal.pone.0056561 (PMC3577874; doi:10.1371/journal.pone.0056561)

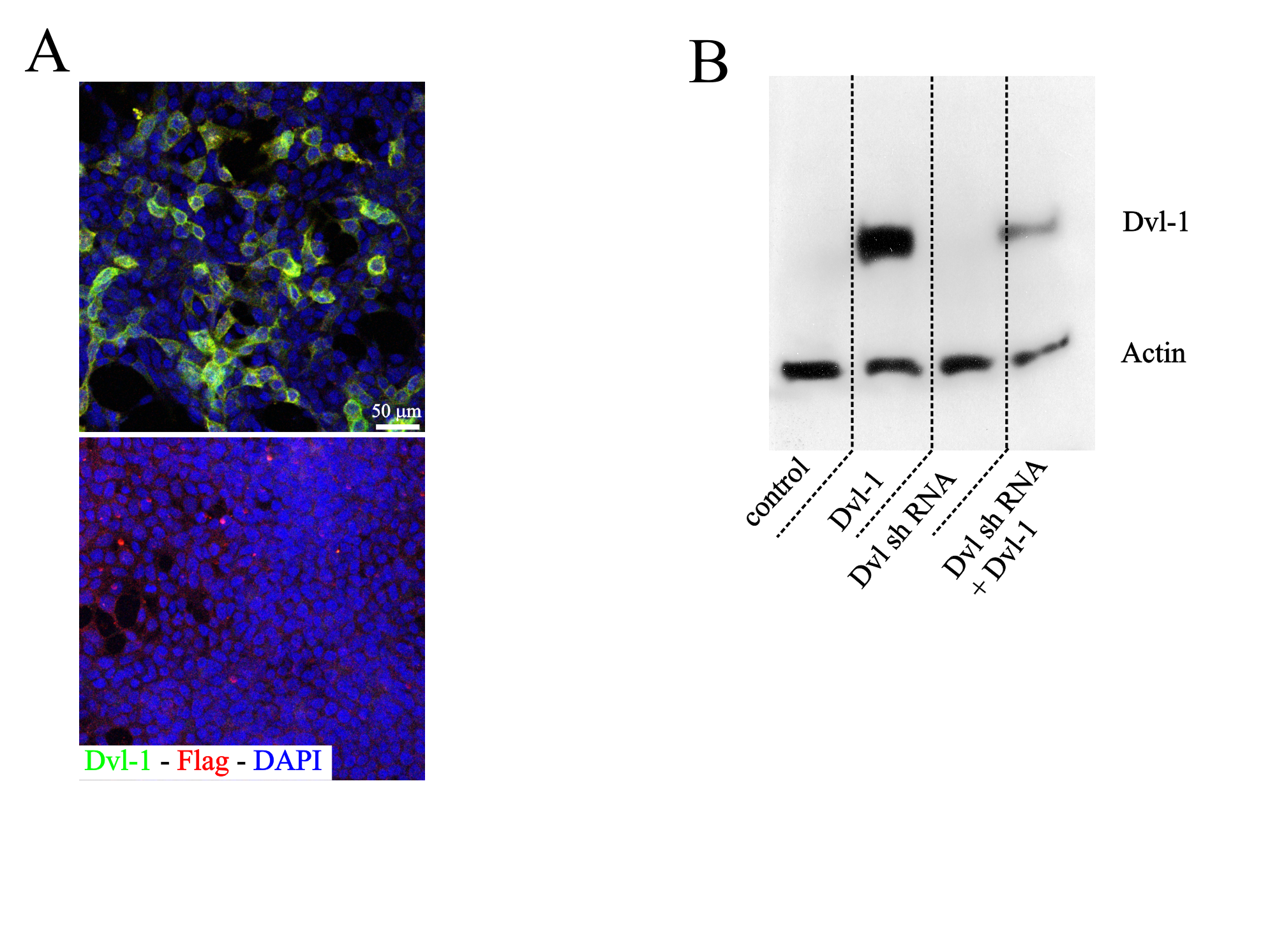

Supplement: Figure S1 — The antibody sc-8025 recognizes Dvl-1 protein. A: COS7 cells were transfected with a Dvl-1 Flag-tagged construct (top) or a control plasmid (bottom) and stained for Dvl-1 (green) and Flag (red), and counterstained for nuclei with Dapi (blue). 100% colocalization was observed between both markers in transfected cells. B: COS7 cells were transfected with the same Dvl-1-Flag plasmid (lanes 2 and 4) in the presence of a Dvl-shRNA (lanes 3 and 4). Homogenized cells were subjected to Western blot analysis and developed with anti-Dvl-1 and anti-β-actin (Abcam) antibodies. Only one specific band of the expected size was observed after transfection with Dvl-1 (lanes 2 and 4) and a reduction of the expression after co-transfection with the Dvl-shRNA (lane 4). Expression levels were standarized dividing Dvl-1 signal by β-actin signal and are shown in Table S1. (TIF) [file pone.0056561.s001.tif]

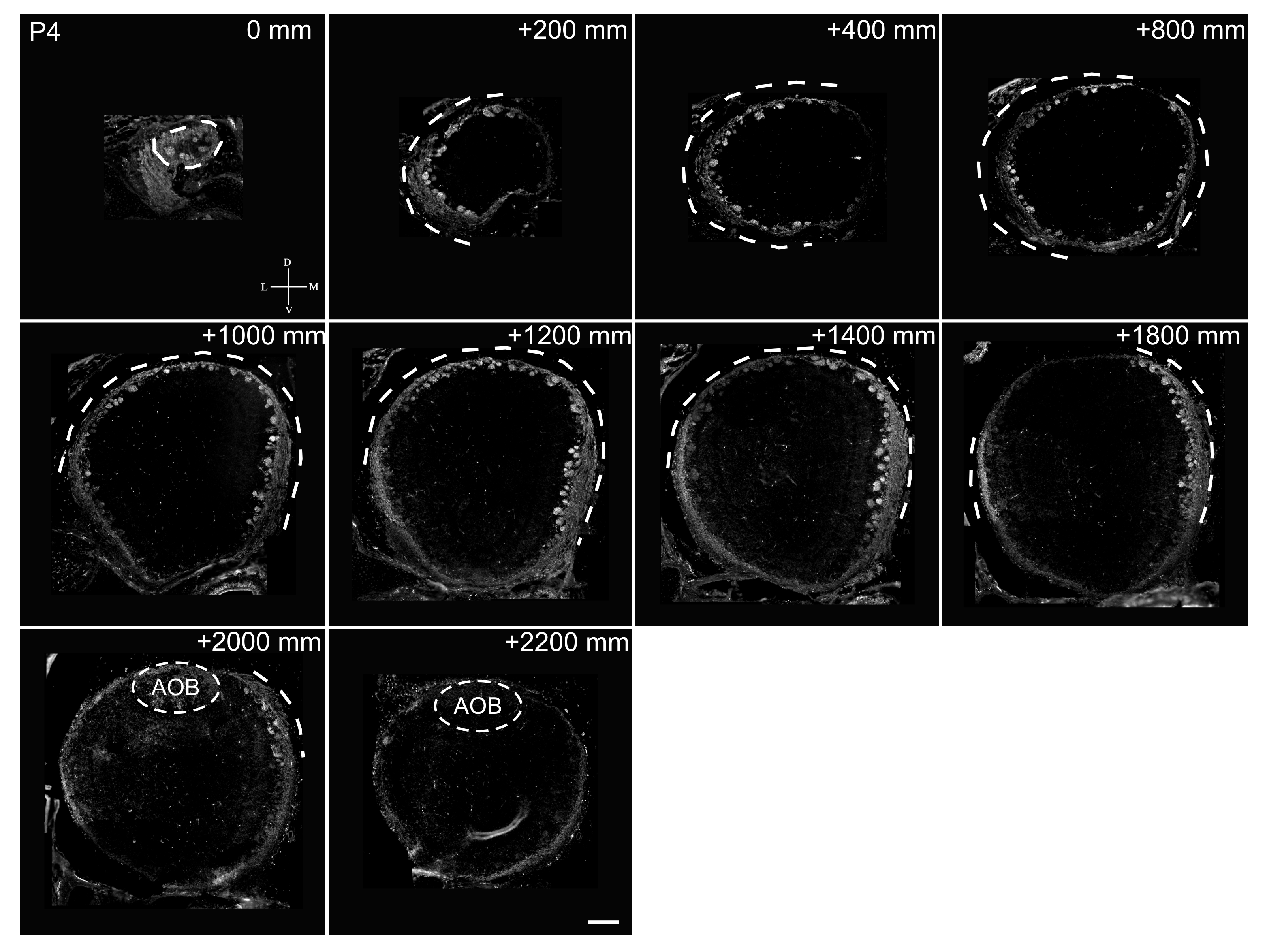

Supplement: Figure S2 — Dvl-1 expression shifts from anterior-lateral to posterior-medial. In agreement with the expression observed in the OE, Dvl-1-expressing axons were observed in a restricted set of glomeruli. At P4 in the most anterior OB sections, dorso-lateral glomeruli showed Dvl-1 expression while posterior sections showed dorso-medial glomeruli Dvl-1 expression. White dashed lines demark where positive Dvl-1 glomeruli were observed. (TIF) [file pone.0056561.s002.tif]

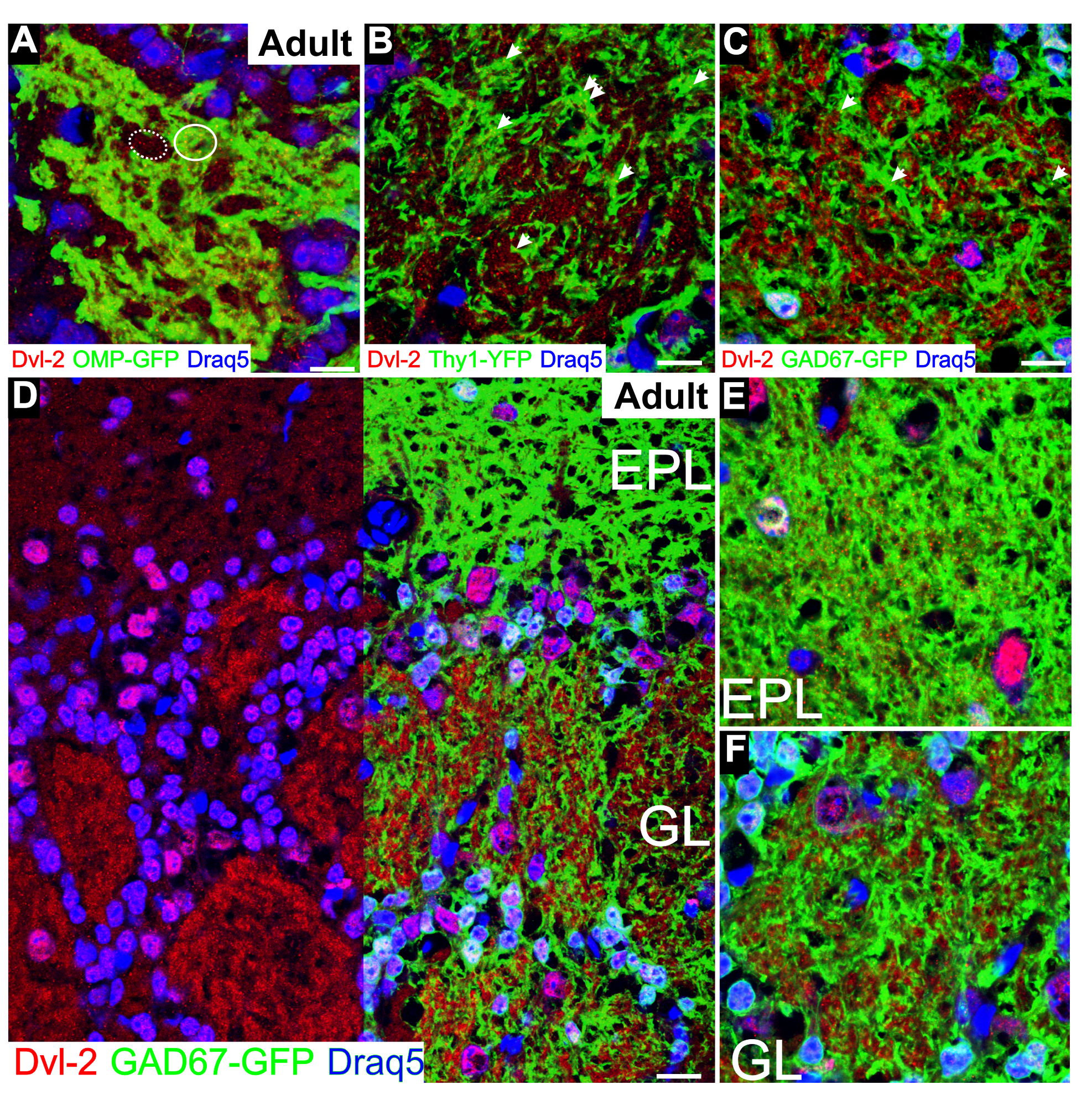

Supplement: Figure S3 — Dvl-2 is observed in OSN axons and dendritic processes in the glomeruli. Most of Dvl-2 puncta was observed in OSN axon, as evidenced for the colocalization in OMP-GFP expressing mice (circle in A).Nonetheless, some of them did not colocalized (dashed circle in A). To analyze which cell type expressed these puncta, we stained for Dvl-2 in Thy1-YFP (to label projection neurons, B) and GAD-67-GFP (to label inhibitory interneurons, C), and in both cases we were able to detect some colocalization (arrows in B and C). Expression of Dvl-2 in the OB of GAD-67-GFP mice showed higher levels of colocalization in the EPL (D, E) than in the glomerular layer (D, F). D: green channel was removed in the left part of the image to show the difference in Dvl-2 expression. Nuclei were counterstained with DRAQ5 (blue). Scale bar = 10 µm in A-C, E, F; 20 µm in D. (TIF) [file pone.0056561.s003.tif]
