# Supplementary material for: Dishevelled Proteins Are Associated with Olfactory Sensory Neuron Presynaptic Terminals
Source: PLoS One. 2013 Feb 20;8(2):e56561. doi: 10.1371/journal.pone.0056561 (PMC3577874; doi:10.1371/journal.pone.0056561)
Supplement: Table S1 — (DOC) [file pone.0056561.s004.doc]

Table S1 (for Supplemental Figure S1):

|  | Dvl-1 / Actin ratio |
| --- | --- |
| Control | --- |
| Dvl-1 | 2.29 |
| Dvl shRNA | --- |
| Dvl sh RNA + Dvl-1 | 0.85 |
